# Content Validity and Psychometric Properties Assessment of the Virtual Reality Functional Capacity Assessment Tool and Application in Multiregional Clinical Trials of Cognitive Impairment Associated with Schizophrenia

**DOI:** 10.1093/schbul/sbag167

**Published:** 2026-10-28

**Authors:** Hans Klein, William P Horan, Nathan Clarke, Fleur M Howells, Kim Cocks, Christina Daskalopoulou, Ben Pascoe, Matthew Reaney, Nicholas Langevin, Justin Corey Fowler, Wenbo Tang, Philip D Harvey, Richard S E Keefe

**Affiliations:** WCG Clinical Endpoints Solutions, Cary, NC 27513, United States; Department of Psychiatry and Biobehavioral Sciences, Jane and Terry Semel Institute for Neuroscience and Human Behavior, UCLA, Los Angeles, CA 90095, United States; Clinical Development, Engrail Therapeutics, San Diego, CA 92130, United States; Patient-Centered Outcomes, Adelphi Values, Bollington, Cheshire SK10 5JB, United Kingdom; Patient-Centred Solutions, IQVIA, London W2 1AF, United Kingdom; Patient-Centered Outcomes, Adelphi Values, Bollington, Cheshire SK10 5JB, United Kingdom; Patient-Centred Solutions, IQVIA, Athens 15232, Greece; Patient-Centered Outcomes, Adelphi Values, Bollington, Cheshire SK10 5JB, United Kingdom; Patient-Centred Solutions, IQVIA, Reading RG1 3JH, United Kingdom; Global Regulatory Strategy, Boehringer Ingelheim Pharmaceuticals Inc., Ridgefield, CT 06877, United States; Clinical Development, Boehringer Ingelheim International GmbH, Ingelheim am Rhein, Mainz-Bingen 55216, Germany; Global Regulatory Strategy, Boehringer Ingelheim Pharmaceuticals Inc., Ridgefield, CT 06877, United States; Department of Psychiatry, University of Miami Miller School of Medicine, Miami, FL 33136, United States; Department of Psychiatry and Behavioral Sciences, Duke University Medical Center, Durham, NC 27710, United States

**Keywords:** cognition, mental health, computer-based, executive function

## Abstract

**Background and Hypothesis:**

Cognitive impairment associated with schizophrenia (CIAS) substantially impacts daily living and is a valuable prognostic indicator of functional outcomes in people living with schizophrenia. The Virtual Reality Functional Capacity Assessment Tool (VRFCAT) is used to assess functional capacity, facilitating the development of new treatments for cognitive impairment. This paper aims to provide evidence supporting the use of the VRFCAT as a valid and reliable performance outcome measure to assess functional change (ie, a fit-for-purpose clinical outcome assessment instrument).

**Study Design:**

This paper is divided into two parts. In Part 1, published and unpublished evidence regarding the suitability of the VRFCAT is synthesized, focusing on content validity, including concept mapping to an updated CIAS conceptual model informed by patient input and evaluation, and known psychometric properties. In Part 2, data collected from three multinational CIAS clinical trials are used to evaluate additional psychometric properties (practice effects, test–retest, and convergent validity) and assess the utility of the VRFCAT within its intended context of use.

**Study Results:**

Published literature supports content validity and key psychometric properties of the VRFCAT. The VRFCAT demonstrates alignment with the signs, symptoms, and impacts in the CIAS conceptual model. Analysis of clinical trial data substantiates published psychometric properties within the intended context of use, with no practice effects identified.

**Conclusions:**

These findings provide evidence that the VRFCAT is a valid and reliable performance outcome measure for use in assessing functional capacity in people with CIAS, supporting its suitability for use in clinical trials.

## Introduction

Cognitive impairment is reported in over 80% of people living with schizophrenia (PLS) and is a core feature of the condition.^1–4^ Signs of cognitive impairment often precede the onset of schizophrenia and include deficits in working and long-term memory, executive function, attention, and higher-order problem-solving.^5–8^ Cognitive impairment associated with schizophrenia (CIAS) is an independent marker for functional outcomes and constitutes a substantial burden affecting all facets of an individual’s life.^5^^,^^6^^,^^9^

Guidance for the evaluation of CIAS recommends the assessment of multiple neurocognitive domains, as identified by the Measurement and Treatment Research to Improve Cognition in Schizophrenia (MATRICS) initiative (2004), including attention/vigilance, working memory, verbal learning, visual learning, reasoning and problem-solving, and social cognition.^10^^,^^11^ A collaboration between the National Institute of Mental Health, the United States Food and Drug Administration (US FDA), and the MATRICS initiative developed guidelines for measuring improvement in CIAS during clinical trials of novel pharmaceutical products. These guidelines also recommend inclusion of instruments capable of assessing real-world functional cognitive improvements^12^; however, no such instrument within the context of a clinical trial has yet been fully established.

Most legacy instruments used to assess cognitive impairment are completed in clinical and laboratory settings. Results of these tests, although valuable, are measures of underlying abilities that are partially correlated with everyday tasks and do not capture the full complexity of real-world functioning.^13^^,^^14^ To address this gap, virtual reality and simulated real-world daily functional activities are increasingly being used to assess cognitive performance when engaging with activities of daily living.^12–15^ Such assessments seek to assess the functional capacity of a participant; in other words, the individual’s ability to complete tasks relevant to daily living. Functional capacity can be defined as the capability to perform activities in a test situation, reflecting the effects of cognition in functioning.^3^ Cognitive impairment may be a precursor to poor functional capacity, which subsequently affects real-world functioning.^10^ Assessment of functional capacity was proposed by the MATRICS initiative as one of two potential co-primary measures of functional outcome for US FDA approval of a neurocognitive drug for schizophrenia.^12^

The Virtual Reality Functional Capacity Assessment Tool (VRFCAT) was developed to measure functional capacity in people living with CIAS in the context of clinical trials.^16^ The overall objective of this paper is to evaluate whether the VRFCAT is fit-for-purpose as a performance outcome (PerfO) measure of functional capacity in people living with CIAS, in alignment with US FDA Patient-Focused Drug Development (PFDD) guidance. To address this objective, the paper is divided into two complementary parts. Part 1 presents a systematic synthesis of published and unpublished evidence regarding the VRFCAT’s content validity, including its placement within a conceptual model of CIAS signs, symptoms, and impacts, and the extent to which the VRFCAT scenarios reflect real-world activities of daily living relevant to functional independence. Part 1 also reviews existing evidence for the VRFCAT’s psychometric properties and construct validity, and includes a new evaluation of alignment with the US FDA PFDD expectations for clinical outcome assessments to identify both strengths and remaining evidence gaps.

Part 2 builds on Part 1’s foundation through a novel psychometric evaluation of the VRFCAT in its intended context of use, using pooled data from three large, multinational, phase 3 clinical trials. Analyses in Part 2 assess feasibility, examine testing (practice) effects, and evaluate test–retest reliability across geographical regions and assessment intervals, as well as replicating and extending construct validity through correlations with cognitive and clinician-rated reference measures and known-groups analyses. Together, Parts 1 and 2 provide a comprehensive, PFDD-aligned evaluation of the VRFCAT’s validity, reliability, and utility for use in multinational CIAS clinical trials.

## Part 1: Synthesis of Existing Evidence Aligned with US FDA PFDD Guidance and Identification of Psychometric Evidence Gaps

### VRFCAT Development and Purpose

The VRFCAT presents simulated scenarios on a digital device, typically a tablet, that require participants to complete a series of 12 tasks or objectives through a progressive storyboard design.^16^^,^^17^ If participants are unable to complete an objective within a prespecified time period or without making a prespecified number of errors, they will be automatically progressed to the next objective.^16^ The 12 tasks or objectives require ~30 min to complete.^18^ Although the process is automated, it is expected to be rater-administered, with trained raters able to assist with technical issues or encourage completion of the assessment. The VRFCAT consists of a tutorial and six versions of four scenarios that have potential to be updated or cross-culturally adapted.^18^

The VRFCAT assesses an individual’s completion of 12 objectives during a virtual shopping trip that incorporates four real-world scenarios: (1) selecting items to create a shopping list, (2) catching the correct bus to the shop, (3) selecting and purchasing items on the shopping list, and (4) paying the correct bus fare to return home.^16^ The VRFCAT developers report that these objectives evaluate several cognitive processes, namely spatial attention, working memory, and executive functioning.^16^^,^^17^ Real-world scenarios that are relevant to functional capacity were incorporated because potential changes in real-world functioning may take longer to present than the duration of typical clinical trials, and may therefore not be observed in a trial setting.^18^

The primary outcome assessment of the VRFCAT is a standardized total score for time taken to complete all 12 objectives. Exploratory outcomes include the number of errors made while attempting each objective and the number of times a participant failed to complete an objective in a 300-second period.^16^ If a participant does not complete an objective in time or makes six errors, they are automatically progressed to the next objective and assigned the maximum score of 300 seconds for the objective.^16^ The total adjusted time for completion is the overall total score for completion of the VRFCAT. This was calculated as the duration of time spent actively working to complete all 12 objectives, excluding time spent receiving automated task instructions and viewing error messages during the assessment. Age- and sex-specific normative data have been collected in a nonclinical population, allowing for calculation of standardized T-scores. There are six VRFCAT parallel forms designed to mitigate potential testing or practice effects and allow for repeated assessment. Each parallel form has the same structure and sequence of objectives, but incorporates variations in recipes, shopping list of items to be purchased (informed by the recipe), bus schedules, and fares.

The following sections provide a comprehensive review and synthesis of the existing evidence regarding the measurement properties of the VRFCAT; a summary of the studies and their primary findings is reported in Table S1. This includes both published literature identified through a search of PubMed and new analyses on the validation dataset shared by the VRFCAT developers.^18^ The alignment of the VRFCAT with the US FDA PFDD clinical outcome assessment guidance document is also considered. Specifically, the review examines the extent to which the VRFCAT fits within conceptual models of CIAS signs, symptoms, and impacts, as well as its ability to examine functional capacity as a measure of real-world activities of daily living. The existing evidence for reliability, structural validity, construct validity, and sensitivity to change is also presented. Finally, the existing evidence is assessed against FDA PFDD expectations for clinical outcome assessments, identifying strengths, and remaining evidence gaps for the VRFCAT.

### VRFCAT within a Conceptual Model of CIAS

The signs, symptoms, and impacts of CIAS were described in a conceptual model by Kitchen and colleagues (2012), which was foundational to understanding the experience of people living with CIAS.^19^ Through primary research with patient advisory boards and workshops, and research conducted by Global Alliance of Mental Illness Advocacy Networks-Europe from 2023 to 2024, the conceptual model by Kitchen et al. was enhanced.^20^ This enhanced version was supported through concept elicitation interviews conducted between 2023 and 2025 with PLS, their caregivers, and healthcare professionals and resulted in several newly identified domains of impact.^20^ The enhanced conceptual model of CIAS articulated the signs, symptoms, and impacts of CIAS, embedding examples of real-world experience derived from the data. Thus, the enhanced conceptual model strongly informs key areas of interest for CIAS research and, of particular relevance to the VRFCAT, identifies key impacts on activities of daily living and independence. Assessments of functional capacity examine the ability of an individual to perform specific skills required for independent living, supporting functional capacity as a concept of interest in CIAS. The specific link between the enhanced conceptual model of CIAS, functional capacity, and the VRFCAT is examined in more detail below.

### VRFCAT as a Measure of Functional Capacity

Establishing content validity involves ensuring that (1) the concept of interest (in this case, functional capacity in people with CIAS) is a relevant and important one, (2) the measure (VRFCAT as a PerfO measure) comprehensively evaluates the concept of interest, and (3) respondents (people living with CIAS) understand the measure and are able to complete the tasks as intended.

#### Relevance and Importance of the Concept of Interest for CIAS

The VRFCAT provides a virtual simulation to measure the functional cognitive impacts of CIAS. These impacts can be linked to deficits in cognitive functioning within schizophrenia across four functional domains aligned to well-documented skill deficits in PLS: meal planning and preparation, use of transportation, shopping, and handling money.^17^^,^^21–24^ Specifically, cognitive impairment has been associated with unhealthy eating habits in schizophrenia, and the challenges associated with planning and preparing nutritious meals manifest in many individuals’ reliance on others to provide their meals. Many PLS face difficulties with shopping for groceries or other items due to challenges in planning, limited shopping strategies (eg, finding the lowest priced item), and difficulty finding the correct items quickly.^17^^,^^23^ PLS often show poor money-management skills, which makes it difficult for them to shop effectively and contributes to the high proportion of individuals who have a trustee to manage their finances.^24^ As a result, PLS are often required to live in residential facilities, where shopping is not necessary, or have family members shop for them.^25^ Finally, PLS are usually less mobile than the general population,^26^ with far fewer having held a driver’s license and many more experiencing challenges related to use of public transportation.^27^ This contributes to a reduced time spent outside of their residence, difficulty accessing healthcare and daily sustenance needs, and greater social isolation. A diagram showing how the VRFCAT maps directly onto the enhanced conceptual model is presented in Figure 1.

**Figure 1:**
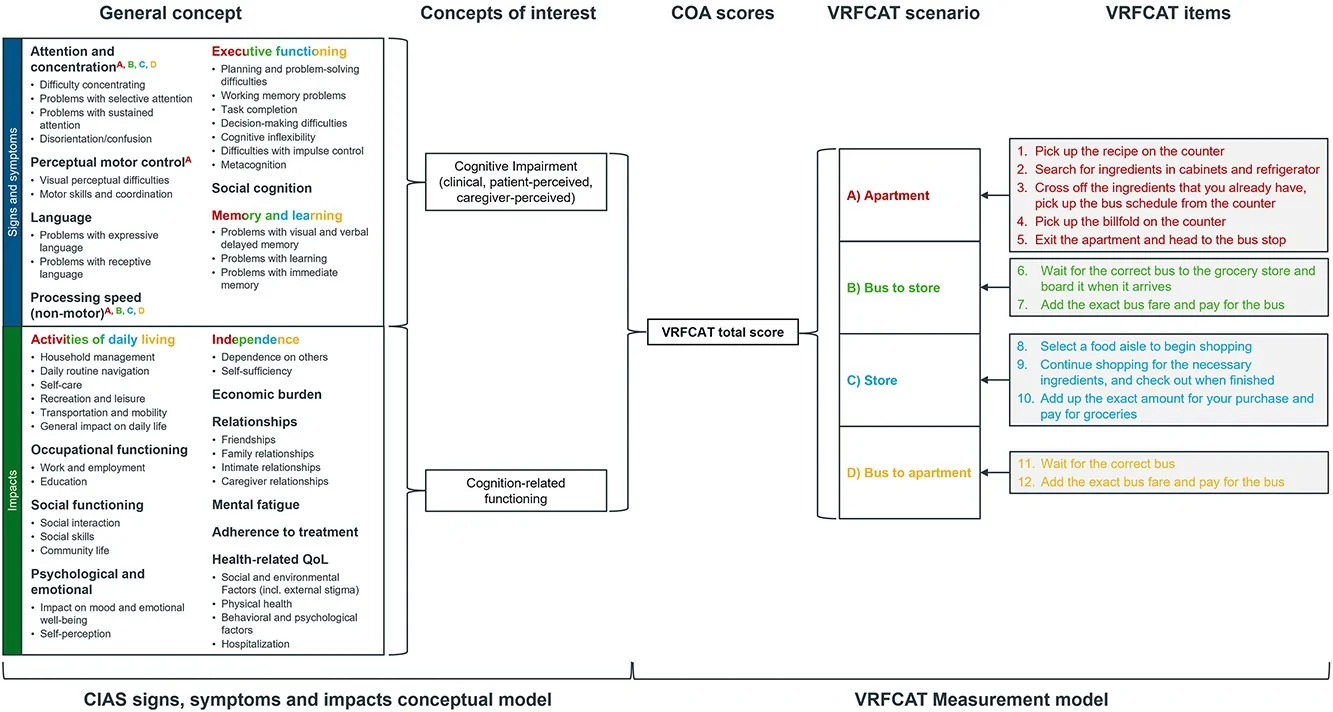
How the VRFCAT Maps onto the Conceptual Model. ^A,B,C,D^ The concept of the item in the COA domain is somewhat related to the concept in the model, showing a connection but not an exact match. CIAS, cognitive impairment associated with schizophrenia; COA, clinical outcome assessment; QoL, quality of life; VRFCAT, Virtual Reality Functional Capacity Assessment Tool

#### Comprehensive Evaluation of Concept of Interest

The four functional skill areas included in the VRFCAT are also represented in other commonly used measures of functional capacity. Since its development in 2001, the University of California San Diego Performance-Based Skills Assessment (UPSA) has been the most extensively used functional capacity measure in schizophrenia research.^28^ Three of the six UPSA skills areas deemed necessary for functioning in the community directly map onto the skills measured by the VRFCAT: household chores (cooking from a recipe and shopping in a grocery store), transportation (use of public transport), and finance (managing money and paying bills). Researchers have consistently demonstrated that performance on these UPSA subtests is substantially impaired in PLS and strongly correlated with global and specific measures of cognition,^29^ as well as with multiple elements of functional independence.^30^ Similarly, another commonly used measure of functional capacity, the Test of Adaptive Behavior in Schizophrenia, includes a shopping skills subtest that involves navigating to a store, using a shopping list, finding items in the store, and correctly paying for the items.^31^ Therefore, the VRFCAT functional skills follow developed instruments, representing functional capacity linked to both cognition and functioning in schizophrenia.

#### Respondent Understanding of the Measure and Ability to Complete Tasks

Regarding the usability of the VRFCAT, the results of a previous qualitative study revealed agreement across key stakeholders that the objectives represent realistic examples of the necessary abilities required for independence within each functional skill area.^17^ Both PLS and caregivers rated VRFCAT tasks as highly relevant and realistic examples of the skills needed for functional independence. Furthermore, PLS described the VRFCAT instructions as easy to understand and the “look and feel” of the VRFCAT as pleasant.^17^

### Existing Evidence for VRFCAT Psychometric Properties

The key psychometric properties identified within the literature are the reliability and validity of the VRFCAT demonstrated within a clinical population. Reliability refers to the degree to which scores are consistent across conditions, which may refer to different sets of items, test forms, or testing occasions. Depending on the conditions, different ways of assessing reliability are available, including internal consistency and test–retest reliability. Evidence relating to the structural and construct validity of the VRFCAT is detailed below.

#### Internal Consistency Reliability

Cronbach’s alpha (*α*) is the proportion of variance in an item set that arises from common variance in the individual items and was used to assess internal consistency of the VRFCAT. Cronbach’s *α* is bounded between 0.0 and 1.0, with higher values indicating a more reliable (homogenous) domain. Acceptable internal consistency reliability is indicated by *α* > 0.70. Although previously unpublished, the internal consistency of the VRFCAT was estimated using the dataset of PLS (*n* = 158) described by Keefe et al. (2016)^18^ and yielded a value of 0.83, indicating acceptable internal consistency across the 12 objectives of the assessment.

#### Test–Retest Reliability

Test–retest reliability is the degree to which a measure produces consistent results over repeated administrations. The test–retest reliability of the VRFCAT total time T-score was examined in Keefe et al. (2016) via an intraclass correlation coefficient (ICC) across two time points 7 to 14 days apart.^18^ The ICC was 0.81 in the PLS group and 0.65 in participants without schizophrenia, indicating a moderate-to-good test–retest reliability for both clinical and nonclinical populations.^18^ Importantly, Keefe et al. (2016) utilized the six alternate forms in their validation work, demonstrating no negative impact of alternate forms on test–retest reliability.^18^

#### Structural Validity

Structural validity is the degree to which the scores of a measurement are an accurate reflection of the dimensionality of the construct being measured. Previous research employed principal components analysis, followed by rotated exploratory factor analysis, to examine the latent structure of the VRFCAT.^32^ The goal was to determine whether a single score could be derived from the VRFCAT or whether multiple scores (ie, multiple factors) would be more appropriate. Analyses were performed separately for participants without schizophrenia (*n* = 165), PLS (*n* = 158), and for the combined group of participants without schizophrenia and PLS, within a multisite observational study.^32^ Both single-factor and multifactorial solutions were examined. Results suggested a unidimensional structure of the VRFCAT across all analyses.

#### Construct Validity

Construct validity is ideally examined through evaluation of the relationship between the score of a measure and additional reference measures of related concepts. This includes convergent/divergent validity analyses using other theoretically related/unrelated measures and known-groups validity analyses, where scores from the target measure are examined to see if they distinguish between groups of individuals that differ by a relevant clinical (or other) indicator of health status or functional capacity.

Within the target population, Keefe et al. (2016) demonstrated moderate-to-strong correlation between the VRFCAT and established reference measures (ie, the UPSA for functional capacity, *r* = 0.58, and the MATRICS Consensus Cognitive Battery [MCCB],^11^  *r* = 0.68).^18^ Similar findings were described for individuals early in the course of schizophrenia (*n* = 42; within 2 years of their first psychotic episode) with absolute values from 0.43 to 0.70 on key reference measures (eg, the UPSA, the MCCB, as well as Cognitive Assessment Interview for cognitive functioning, and a real-world functional measure: the Global Functioning Scale: Role and Social).^33^ Comparing overall performance on the VRFCAT between clinical and nonclinical groups, Keefe et al. (2016) demonstrated that participants without schizophrenia (*n* = 165) had a mean T-score of 49.7 compared with a T-score of 32.5 in PLS (*n* = 158); Cohen’s *d* was 1.21, indicating a large effect of condition on performance and providing support of known-groups validity.^18^ Similarly, findings were again described for those early in the course of schizophrenia when compared with participants without schizophrenia (*n* = 13). PLS in its early course performed slower than participants without schizophrenia (830.41 vs 716.84 s; *P* < .01), further supporting known-groups validity at onset of the illness.^33^

#### VRFCAT Sensitivity to Change

Evidence of sensitivity to change was provided from a study examining the impact of an online, plasticity-based training program that targets social cognition deficits in schizophrenia.^34^ In the study, clinically stable PLS were randomly assigned to either experimental (social cognition training) or active control (casual games) conditions and completed training sessions for 8–12 weeks. The VRFCAT total score was a secondary outcome in the study and significantly improved following the experimental intervention (linear mixed-effects model: *b* = −57.53, |*z*| = 2.24, *P* = .025); however, this effect was not statistically significant after Bonferroni correction for multiple testing.^34^

### VRFCAT as a Clinical Outcome Assessment and Alignment with Regulatory Expectations

Existing evidence suggests that the VRFCAT is a reliable and valid assessment of functional capacity, offering a more holistic assessment of cognitive performance across a range of tasks within a realistic scenario. The objective scoring of the VRFCAT incorporates both response time and accuracy in capturing functional capacity. Given that the VRFCAT assesses functional capacity at the time of measurement, it does not require the participant to recall any information and thus avoids biases that may impact interviews of functional cognition. Published data demonstrate limited practice effects, acceptable test–retest reliability and, as noted in a comparative study, VRFCAT normed adjusted total time T-score correlated significantly with MCCB and UPSA scores and provided superior sensitivity in detecting functional deficits.^33^

The US FDA PFDD guidance strongly suggests the incorporation of patient experience during the development of PerfO measures.^35^ This approach supports creating the necessary linkage between a task’s measurement (eg, total adjusted time) and real-life daily impacts associated with CIAS. The VRFCAT developers have conducted primary research through interviews with people living with CIAS and have shown through modeling that functional capacity, the primary measurement of the VRFCAT, mediates the relationship between neurocognition and real-world community functioning.^17^ Furthermore, qualitative feedback from participants indicates the “look and feel” of the VRFCAT to be pleasant and the instructions easy to understand.^17^ Combined with the ease of administration, this supports the utilization of the VRFCAT in people living with CIAS. However, critical to the published literature to date, validation work with the VRFCAT has primarily taken place in academic centers in the United States, with limited published information on how the scale performs within its intended context of use in multinational clinical trials. In Part 2, the reliability and validity of the VRFCAT is examined as implemented in a multinational, phase 3 clinical program.

## Part 2: Psychometric Evaluation of VRFCAT within the CONNEX Clinical Trials

### Methods

The primary dataset utilized for the psychometric evaluation of the VRFCAT came from the three pivotal, multinational, randomized, double-blind, placebo-controlled, phase 3 clinical trials: CONNEX-1 (NCT04846868), CONNEX-2 (NCT04846881), and CONNEX-3 (NCT04860830). These trials evaluated the efficacy and safety of once-daily iclepertin in PLS over 26 weeks (Figure S1). Enrollment in the CONNEX-1, -2, and -3 trials comprised adult (aged 18-50 years), clinically stable PLS in the residual (non-acute) phase of their illness, with functional impairment in day-to-day activities, per investigator judgment.

The six versions of the VRFCAT were counterbalanced across visits and sites to reduce version effects and ensure no participant received the same version more than once in the CONNEX-1, -2, and -3 trials. The VRFCAT was administered at screening, baseline, Week 12, and Week 26.

Unless otherwise noted, VRFCAT results were evaluated using an interim, pooled, blinded data cut that included all participants who completed key study endpoints (ie, VRFCAT, MCCB, Schizophrenia Cognition Rating Scale [SCoRS], or Patient-Reported Experience of Cognitive Impairment in Schizophrenia [PRECIS]) during screening and at baseline. Note that analysis of changes over time included participants who had completed the specified follow-up visit at the time of the data cut (August 23, 2024 for the CONNEX-1 and CONNEX-2 trials, and September 3, 2024 for the CONNEX-3 trial).

### Feasibility and Descriptive Analyses

Feasibility of administration within a multinational clinical program was evaluated via missing data rates, specifically the number and percentage of VRFCAT scores not available relative to the number of completed assessments for participants still enrolled in the trial at each assessment time point. Descriptive analyses, including total time T-score, error counts, and forced progressions, were examined at each available time point.

### VRFCAT Reliability

#### Testing (Practice) Effects

To examine potential effects from repeated administration of the VRFCAT, paired *t*-tests were used to evaluate the statistical significance and effect sizes of changes in the VRFCAT. Screening and baseline assessments (approximately 4–5 weeks between testing) were used for these analyses to reduce the potential impact of interventional treatment on participants’ scores. Cohen’s *d* was used as the measure of effect size, which can be interpreted using the following thresholds: small (effect size ≥0.20 to <0.50), moderate (effect size ≥0.50 to <0.80), and large (effect size ≥0.80).^36^

#### Test–Retest Reliability

Test–retest reliability was evaluated within the dataset in two distinct ways. First, pretreatment test–retest reliability (approximately 4-5 weeks between assessments) was assessed for the global pooled sample, with additional ad hoc regional evaluation of test–retest reliability explored for the largest regional subsamples (China, Europe, Japan, Latin America, and the United States). Second, longer-term (3 month) test–retest reliability was estimated within subsamples of the blinded dataset. Subsampling was employed to reduce the potential impact of either interventional drug therapeutic effects or deteriorating psychosis on estimates of long-term reliability, and three groups were defined based on these concerns. Long-term test–retest reliability was estimated for participants who met the following criteria: (1) Positive and Negative Syndrome Scale that changed less than 10 points between screening and baseline, (2) no change on a single item on the SCoRS asking about changes since starting study treatment at Week 12, or (3) a Clinical Global Impression of Severity (CGI-S) level change of zero from baseline to Week 12 (Table S2). All test–retest reliability estimates were calculated with the ICC based on a single-measurement, absolute agreement, two-way mixed-effects model.

#### Construct Validity

Construct validity was explored by evaluating the relationships between the VRFCAT and the reference measures from the CONNEX trials. As part of the exploratory analyses to replicate convergent analyses from the initial validation work for the VRFCAT,^18^ cross-sectional correlations were examined between the VRFCAT adjusted total time T-score and the MCCB overall composite T-score, SCoRS interviewer total score, and PRECIS total score using the CONNEX-1, -2, and -3 dataset. According to previous research, these measures were expected to be correlated between 0.40 and 0.80,^37^ and correlations between the VRFCAT and MCCB were expected to be stronger than between the VRFCAT and SCoRS or between the VRFCAT and PRECIS due to method variance.

To examine the VRFCAT’s known-group validity within a clinical population, participants were categorized at each available time point based on CGI-S level (CGI-S terminology and rating levels provided in Table S3). VRFCAT performance within each rating category was descriptively summarized, including the number of participants, mean, standard deviation (SD), median, minimum, and maximum values (Table 1). Between-group differences were evaluated using effect size estimates.

**Table 1:** Descriptive Statistics for Pooled CONNEX-1, -2, and -3 Data

	Screening				Baseline				Week 12				Week 26			
	*N*	Mean (SD)	Median	Range	*N*	Mean (SD)	Median	Range	*N*	Mean (SD)	Median	Range	*N*	Mean (SD)	Median	Range
**VRFCAT total adjusted time T-score**	1763	35.6 (20.36)	41.1	−102, 66	1798	34.8 (20.34)	39.8	−101, 65	1681	36.2 (20.19)	41.9	−75, 67	1374	36.5 (21.98)	43.0	−121, 63
**VRFCAT total errors T-score**	1763	42.1 (21.79)	49.0	−131, 60	1798	40.8 (23.00)	48.1	−138, 60	1681	41.4 (21.47)	48.1	−146, 60	1374	40.5 (25.66)	48.9	−248, 60
**VRFCAT total forced progressions T-score**	1763	39.0 (32.16)	52.3	−310, 54	1798	39.1 (31.17)	52.3	−272, 54	1681	39.9 (30.84)	52.3	−182, 54	1374	39.4 (34.97)	52.3	−345, 54

Abbreviations: SD, standard deviation; VRFCAT, Virtual Reality Functional Capacity Assessment Tool.

Statistical significance (*P* ≤ .05) of between-group differences was assessed using a one-way analysis of variance (ANOVA) with *F*-tests, followed by Tukey’s post hoc test for comparisons involving three or more groups and between-group pairwise comparisons. Additionally, Jonckheere–Terpstra test statistics were used to evaluate monotonic trends in the scores across ordered known groups.^38^ Evidence supporting known-groups’ validity was based on a logical pattern of mean differences and corresponding effect sizes, with statistical significance tests providing supplementary support.

## Results

### Feasibility and Descriptive Analyses

The form-level missing data rate for the VRFCAT was low, with rates at 3.9% (72/1835) for screening, 2.0% (37/1835) at baseline, 2.2% (37/1717) at Week 12, and 3.6% (49/1349) at Week 26. At screening and baseline, the normalized total time T-score was approximately 35, with Weeks 12 and 26 nominally higher around 36. Across time points, there were notable outliers observed within the data with minimum T-scores well below zero, while the maximum score recorded was >60.

### Evaluation of Psychometric Properties

#### Testing (Practice) Effects

Practice effects were assessed across all participants in the pooled data from the CONNEX-1, -2, and -3 trials. The VRFCAT adjusted total time T-scores at screening and baseline were included in the analysis to determine the magnitude of change before study drug administration (*n* = 1727). Practice effects in the pooled sample were small, with the adjusted total time T-score (SD) decreasing from screening to baseline by −1.0 (SD = 13.87) points, an improvement equal to Cohen’s *d* of −0.05.

#### Test–Retest Reliability

Pretreatment test–retest reliability of the VRFCAT demonstrated overall good reliability (ICC = 0.779) across the study screening period globally, with regional reliability estimates largely in line (Table 2). Longer-term test–retest reliability (baseline to Week 12) estimates within each of the three post-treatment stability subsamples demonstrated similarly “good” reliability (ICC > 0.75) (Table S4).

**Table 2:** Test–Retest Reliability of the VRFCAT Adjusted Total Time T-Score (Pooled Data from CONNEX-1, -2, and -3)

	Screening			Baseline			Practice effects and test–retest		
Region	*n*	Mean (T-score)	SD	*n*	Mean (T-score)	SD	*T*-test (*P* value)	Cohen’s *d*	ICC
Global	1779	35.82	20.23	1779	35.07	19.95	2.375 (.018)	0.056	0.779
United States	255	41.00	16.15	255	40.03	15.84	1.224 (.222)	0.077	0.687
Japan	164	47.69	13.46	164	48.35	12.17	−0.998 (.320)	0.078	0.785
China	215	33.01	17.94	215	32.31	16.81	0.727 (.468)	0.050	0.669
Latin America	546	27.57	23.01	576	26.64	22.45	1.409 (.159)	0.059	0.756
Europe	409	39.68	18.54	409	39.14	18.51	0.911 (.363)	0.045	0.787

Abbreviations: ICC, intraclass correlation coefficient; SD, standard deviation; VRFCAT, Virtual Reality Functional Capacity Assessment Tool.

### Construct Validity

As expected based on published results, correlations between the VRFCAT and MCCB were larger (*r* > 0.60) than between the VRFCAT and SCoRS (|*r*| ≈ 0.30) and between VRFCAT and PRECIS (|*r*| ≈ 0.10) (Table 3). Results were also largely replicated across regions (Table S5).

**Table 3:** Evaluation of Convergent Validity for VRFCAT: Pearson’s Correlation (Absolute Value) of VRFCAT to Other CIAS Clinical Outcome Assessments

	Pearson correlation coefficient (*N*)		
	MCCB overall composite T-score	SCoRS interviewer total score	PRECIS
Baseline	0.65 (1831)	0.26 (1832)	0.09 (1828)
Week 26	0.67 (1606)	0.29 (1597)	0.11 (1600)

Abbreviations: CIAS, Cognitive Impairment Associated with Schizophrenia; MATRICS, Measurement and Treatment Research to Improve Cognition in Schizophrenia; MCCB, MATRICS Consensus Cognitive Battery; PRECIS, Patient-Reported Experience of Cognitive Impairment in Schizophrenia; SCoRS, Schizophrenia Cognition Rating Scale; VRFCAT, Virtual Reality Functional Capacity Assessment Tool.

#### Known-Groups Validity

Known-groups validity based on severity groupings within the CGI-S level at baseline, Week 12, and Week 26 (Table 4 and Figures S2-S4) revealed overall separation of VRFCAT performance for participants rated “mild,” “moderate,” and “severe,” with more severe symptoms relating to poorer performance on the assessment. Comparisons between “none” and “mild-severity” groups yielded negligible effect sizes or lacked statistically significant difference, potentially due to small sample sizes; however, overall results supported known-groups validity, with significant linear trends, ANOVA results, and monotonicity of mean and median scores being demonstrated at each time point assessed.

**Table 4:** Summary of Known-Groups Validity for Baseline, Week 12, and Week 26

	*N*	Mean (SD)	Median (min, max)	Between-groups comparisons (*P* value)	Between-groups effect size	Linear trend (*P* value)	*F*-test (*P* value)
**CGI-S levels**							
**Baseline**							
None	26	41.50 (16.08)	45.40 (−21.01, 58.71)			<.001	<.001
Mild	706	39.58 (16.84)	43.63 (−66.27, 64.71)	.963	−0.114		
Moderate	929	31.91 (20.50)	36.33 (−64.78, 63.97)	.073	−0.470		
Severe	90	21.37 (32.54)	32.80 (−100.88, 60.70)	<.001	−0.677		
**Week 12**							
None	70	42.44 (12.54)	44.61 (1.59, 67.26)			<.001	<.001
Mild	937	39.59 (17.55)	44.09 (−61.56, 65.56)	.635	−0.165		
Moderate	631	31.79 (21.39)	37.48 (−72.00, 62.24)	<.001	−0.515		
Severe	30	3.35 (34.75)	11.14 (−74.62, 54.57)	<.001	−1.807		
**Week 26**							
None	94	44.17 (21.96)	49.19 (−110.88, 61.43)			<.001	<.001
Mild	841	39.88 (17.61)	44.68 (−60.28, 63.48)	.226	−0.237		
Moderate	386	30.47 (24.25)	37.35 (−121.20, 62.15)	<.001	−0.575		
Severe	27	−7.92 (41.30)	−1.95 (−79.72, 54.68)	<.001	−1.903		

Abbreviations: CGI-S, Clinical Global Impression of Severity; max, maximum; min, minimum; SD, standard deviation.

## Discussion

The dual aims of this paper were to synthesize available evidence supporting use of the VRFCAT as a potential fit-for-purpose PerfO measure and to leverage pooled data from the CONNEX clinical program to evaluate the psychometric properties of the VRFCAT within its intended context of use. Consequently, the paper provides robust evidence supporting the reliability, validity, and clinical relevance of the VRFCAT in people with CIAS.

Part 1 demonstrates that published research reports the VRFCAT to adequately assess skills that are viewed as relevant and important to key stakeholders, similar to previously developed assessments of functional capacity. Much of the qualitative research that provides strong evidence for the content validity of the VRFCAT was generated after its initial development. The findings indicate that these skills are impacted by CIAS and potentially limit the ability for people with CIAS to achieve independence. Further, research has demonstrated that the VRFCAT is reliable upon repeated testing, adequately discriminates between healthy and impacted individuals, and is sensitive to intervention.

In line with the US FDA PFDD guidance, it is crucial to demonstrate the VRFCAT’s psychometric properties in a sample within its target population and in the context of an international clinical trial. Part 2 evaluated the psychometric properties of the VRFCAT within a multinational clinical development program. The VRFCAT demonstrated strong internal consistency (*α* = 0.83) and moderate-to-good test–retest reliability globally at 4–5-week and 12-week intervals (ICC > 0.75). Construct validity was confirmed by moderate-to-strong correlations with established cognitive and functional measures (MCCB and UPSA), and known-groups validity was shown through differences in scores across CGI-S levels. The VRFCAT demonstrated minimal practice effects (Cohen’s *d* = −0.05) and psychometric properties were largely consistent despite regional differences in the overall level of impairment, as assessed by the VRFCAT and MCCB. Compared with the MCCB, construct validity analysis showed weaker correlations between the VRFCAT and SCoRS, and particularly PRECIS. While this may likely reflect method variance, the weaker correlation with patient-reported cognitive impairment may raise questions about the relationship between objective functional capacity and subjective cognitive experience. There is also the possibility of less reliable self-reporting of real-world functioning due to reporting bias and impaired introspective accuracy, with PLS potentially misjudging their cognition and functioning.^39^ Overall, the results of Part 2 expand the evidence on reliability of the VRFCAT, evaluate its performance across large, multinational clinical trials, and examine stability and practice effects across geographic regions.

Despite the strengths of the current work, there are limitations that future research should consider. Primarily, the psychometric evaluations focused on the total adjusted time score, with limited exploration of other scoring dimensions. Validation work on the scale suggested that the total time to completion was the most sensitive to known-group differences and thus should be the primary endpoint^18^; however, secondary scoring metrics, such as error and forced progression counts, could provide meaningful context for overall performance and understanding extreme outlier performance, although this has not been explored to date. Descriptive statistics for pooled CONNEX-1, -2, and -3 data highlighted minimum total adjusted time T-score below −100, representing participants who took much longer than expected to complete the tasks, relative to an individual without schizophrenia. One potential explanation for this is misunderstanding of the task instructions. This could be compounded by inattention or errors on the part of the examiner. Future research could utilize these data to propose a priori cut-off values for exclusion criteria, using the number of forced progressions within the VRFCAT as a proxy for both participants’ understanding of the task, as well as rater feedback on participant effort, ability to perform the task, and examiners’ supervision of their performance. Finally, while content validity analyses indicated a generally high level of perceived relevance and realistic examples of skills needed for functional independence,^17^ there is quite likely to be a difference between ability in a simulated virtual reality scenario and performance measured in a real-world setting. Ongoing work is exploring other aspects of the VRFCAT that could aid in identifying responders to treatment, including identification of thresholds for meaningful change, and examination of sensitivity to pharmacologic treatment. These efforts will supplement the data presented here as well as explore the other key endpoints within the CONNEX dataset.

In conclusion, the findings from this study provide substantial support for the VRFCAT moving toward becoming a fit-for-purpose PerfO measure of functional capacity in CIAS. The VRFCAT measures domains that directly impact PLS and is understandable and usable by respondents. Its strong psychometric profile and clinical relevance make it a valuable tool for evaluating treatment effects and functional outcomes in schizophrenia research. Continued refinement and validation, including regulatory alignment, will further strengthen its utility in advancing treatment evaluation and improving outcomes for PLS.

## Supplementary Material

Supplementary_materials_sbag167

## Data Availability

All data generated or analyzed during this study, which support the findings of this study, are included within this article and its supplementary information files. Researchers interested in further analyses not presented in the manuscript may contact the corresponding author.
