# Supplementary material for: Content Validity and Psychometric Properties Assessment of the Virtual Reality Functional Capacity Assessment Tool and Application in Multiregional Clinical Trials of Cognitive Impairment Associated with Schizophrenia
Source: Schizophr Bull. 2026 Oct 28;52(6):sbag167. doi: 10.1093/schbul/sbag167 (PMC13716497; doi:10.1093/schbul/sbag167)
Supplement: Supplementary file 1 [file supplementary_materials_sbag167.docx]

# Supplementary Materials

## Table S1. Summary of existing and novel evidence for the VRFCAT as a potential fit-for-purpose assessment

| **Concept evaluated (adapted from FDA COA Qualification Packages Outline)** | **Citation (if applicable)** | **Study population** | **Finding** |
| --- | --- | --- | --- |
| Concept of interest is of meaningful treatment benefit | Langevin, et al (2026) | PLS (n=26)  Healthcare stakeholders (n=83)  Patient advisory board members (n=32)  Clinical trial exit interviews from the CONNEX trial (n=37) | Updated conceptual the model of CIAS, including key impacts on activities of daily living and independence, aligned with the concept of functional capacity |
| Expert input/respondent input to support that the assessment is representative of the concept of interest and is relevant to the ability to function in day-to-day life | Horan, et al (2023) | PLS (n=24)  Caregivers (n=12)  Professional peer-support specialists (n=12) | The VRFCAT was viewed as relevant, and meaningful examples of the four functional skill domains were targeted by the assessment |
| Dimensionality analysis | Harvey, et al (2020) | PLS (n=158)  Healthy participants (n=165) | Results suggested a unidimensional structure of the VRFCAT across all analyses |
| Feasibility | Keefe, et al (2016) | PLS (n=158)  Healthy participants (n=166) | Participant reports found the task to be pleasant to take, easy to use, and found instructions clear |
|  | Not previously published | As described in the manuscript | Overall low missingness rate (<4% at each testing timepoint) |
| Testing (practice) effects | Keefe, et al (2016) | PLS (n=158)  Healthy participants (n=166) | When tested 7 to 14 days apart, minimal effects observed (Cohen’s d=−0.04) |
|  | Not previously published | As described in the manuscript | When tested 4 to 5 weeks apart, minimal testing effects observed (Cohen’s d=−0.05) |
|  |  |  | Regional subgroups similarly demonstrated minimal testing effects (Cohen’s d between 0.045 and 0.078) |
| Test–retest reliability analysis^a^ | Keefe, et al (2016) | PLS (n=158)  Healthy participants (n=166) | Good reliability for PLS (ICC=0.81); moderate for healthy participants (ICC=0.65) |
|  | Not previously published | As described in the manuscript | When tested 4 to 5 weeks apart (pre-treatment) good reliability (ICC=0.779) |
|  |  |  | Regional subgroups demonstrated moderate to good reliability (ICCs between 0.669 and 0.787) |
|  |  |  | When tested around 12 weeks later, symptom-stable subgroups demonstrate good reliability (ICCs between 0.792 and 0.765) |
| Internal consistency reliability analysis | Not previously published | Novel results utilizing dataset from Keefe, et al (2016) VRFCAT validation paper  PLS (n=158)  Healthy participants (n=166) | Acceptable internal consistency (Cronbach’s alpha = 0.83) |
| Convergent and discriminant validity analysis (i.e., association with other instruments assessing similar or different concepts) | Keefe, et al (2016) | PLS (n=158) | In PLS, VRFCAT demonstrated moderate correlations with UPSA (r=0.58) and MCCB (r=0.68). VRFCAT demonstrated weaker correlations with SCoRS (r=0.29) |
|  | Ventura, et al (2020) | PLS, early disease course (n=42)  Healthy participants (n=13) | In PLS, VRFCAT demonstrated moderate correlations with UPSA (r=0.66), MCCB (r=0.70), CAI (r=0.51), and GFS (r=0.52) |
|  | Not previously published | As described in manuscript | VRFCAT demonstrated moderate correlations with MCCB at baseline (r=0.65) and Week 26 (r=0.67). VRFCAT demonstrated weaker correlations with SCoRS and PRECIS at baseline (r=0.26 and r=0.09, respectively) as well as Week 26 (r=0.29 and r=0.11 respectively) |
| Known-groups validity analysis (e.g., difference in scores between subgroups of subjects with known status) | Keefe, et al (2016) | PLS (n=158)  Healthy participants (n=166) | Healthy participants’ mean T-score = 49.7 compared with PLS T-score = 32.5 (Cohen’s d=1.21) |
|  | Ventura, et al (2020) | PLS, early disease course (n=42)  Healthy participants (n=13) | Healthy participants performed task faster (716.84 seconds) compared with PLS early disease course (830.41 seconds) |
|  | Not previously published | As described in manuscript | When looking at levels of severity based on CGI-S, between-group differences seen for mild, moderate, and severe participants across baseline, Week 12, and Week 26 time points (effect sizes ranging from −0.470 to −1.903) |
| Results from the evaluation of the instrument’s ability to detect change^b^ | Nahum M, et al (2021) | PLS: n=147, 55 completers in the intervention arm | VRFCAT total score significantly improved following the experimental intervention (linear mixed-effects model: b=−57.53, \|z\|=2.24, P=0.025); however, this effect was not statistically significant after Bonferroni correction for multiple testing |
| Evaluation and definition of meaningful within person change (improvement and worsening)^b^ |  |  |  |
| Brief aims of cited articles:  Langevin, et al (2026): Targeted literature review and concept elicitation of key stakeholders identifying domains impacting PLS's daily life and independent living;  Horan, et al (2023): Individual, semi-structured interviews of three key stakeholder groups assessing the importance and relevance of the targeted domains of the VRFCAT to independent functioning;  Harvey, et al (2020): Utilized dataset from the Keefe, et al (2016) VRFCAT validation paper to assess factor structure of the VRFCAT assessment;  Keefe, et al (2016): Original VRFCAT psychometric validation paper;  Ventura, et al (2020): VRFCAT validation paper for use with PLS early in course of illness (within 2 years of their first psychotic episode);  Nahum, et al (2021): Clinical trial assessing impact of online training program on social cognition in schizophrenia; VRFCAT was a secondary outcome included in the trial. | | | |

^a^As six alternate forms were utilized to estimate value in both Keefe, et al (2016) and CONNEX, these results similarly demonstrate no negative impact of alternate forms on test–retest reliability.

^b^Concepts identified as requiring additional support.

CAI, Cognitive Assessment Interview; CGI-S, Clinical Global Impression of Severity; CIAS, cognitive impairment associated with schizophrenia; COA, clinical outcome assessment; FDA, US Food and Drug Administration; GFS, Global Functioning Scale; ICC, intraclass correlation coefficient; MATRICS, Measurement and Treatment Research to Improve Cognition in Schizophrenia; MCCB, MATRICS Consensus Cognitive Battery; PLS, people living with schizophrenia; PRECIS, Patient-Reported Experience of Cognitive Impairment in Schizophrenia; SCoRS, Schizophrenia Cognition Rating Scale; UPSA, University of California San Diego Performance-based Skills Assessment; VRFCAT, Virtual Reality Functional Capacity Assessment Tool.

## Table S2. Definitions of stability

| Time point | Measure of stability | Definition of stability |
| --- | --- | --- |
| Screening to baseline | PANSS | PANSS change = ±10 |
| Baseline to Week 12 | SCoRS patient change rating | No change |
| Baseline to Week 12 | CGI-S | Change in CGI-S level = 0 |

CGI-S, Clinical Global Impression of Severity; PANSS, Positive and Negative Syndrome Scale; SCoRS, Schizophrenia Cognition Rating Scale.

## Table S3. Known-group categories and rationale for selection of supportive measure and time points for assessment

| Known-group definitions (supporting measure) | Time points to be evaluated | Rationale |
| --- | --- | --- |
| CGI-S level:   - None - Mild - Moderate - Severe | • Baseline  • Week 12  • Week 26 | CGI-S raters consider the totality of evidence, including other clinical outcome assessments, to derive a global rating and have clinical expertise |

CGI-S, Clinical Global Impression of Severity; VRFCAT, Virtual Reality Functional Capacity Assessment Tool.

## Table S4. Test–retest reliability of the VRFCAT adjusted total time T-score (CONNEX-1, -2, and -3)

| **Clinical outcome assessment score/anchor stability definition** | **Time points** | **Psychometric analysis population (N=1339)** | |
| --- | --- | --- | --- |
|  |  | **N** | **ICC estimate (95% CI)** |
| **PANSS (change ≤ 10)** | Screening to baseline | 1407 | 0.765 (0.742, 0.786) |
| **SCoRS patient change rating (change = 0)** | Week 12 | 562 | 0.793 (0.760, 0.822) |
| **CGI-S (CGI-S level = 0)** | Baseline to Week 12 | 1096 | 0.792 (0.769, 0.814) |

CI, confidence interval; CGI-S, Clinical Global Impression of Severity; ICC, intraclass correlation coefficient; PANSS, Positive and Negative Syndrome Scale; SCoRS, Schizophrenia Cognition Rating Scale; VRFCAT, Virtual Reality Functional Capacity Assessment Tool.

## Table S5. Correlations between VRFCAT adjusted total time T-scores and reference measures across regions (pooled data from CONNEX-1, -2, and -3)

| **Region** | **Visit** | **MCCB overall composite T-score** | | **SCoRS interviewer total score** | |
| --- | --- | --- | --- | --- | --- |
|  |  | **n** | **Pearson correlation coefficient** | **n** | **Pearson correlation coefficient** |
| Global | Screening | 1768 | 0.609 | 1762 | −0.270 |
|  | Baseline | 1668 | 0.649 | 1791 | −0.257 |
| United States | Screening | 251 | 0.552 | 247 | −0.167 |
|  | Baseline | 242 | 0.535 | 253 | −0.200 |
| Japan | Screening | 158 | 0.579 | 158 | −0.105 |
|  | Baseline | 163 | 0.597 | 165 | −0.075 |
| China | Screening | 207 | 0.584 | 207 | −0.298 |
|  | Baseline | 191 | 0.581 | 215 | −0.339 |
| Latin America | Screening | 591 | 0.533 | 590 | −0.213 |
|  | Baseline | 526 | 0.652 | 575 | −0.225 |
| Europe | Screening | 406 | 0.602 | 405 | −0.302 |
|  | Baseline | 390 | 0.589 | 421 | −0.236 |

MATRICS, Measurement and Treatment Research to Improve Cognition in Schizophrenia; MCCB, MATRICS Consensus Cognitive Battery; SCoRS, Schizophrenia Cognition Rating Scale; VRFCAT, Virtual Reality Functional Capacity Assessment Tool.

## Figure S1. CONNEX trials study design^a^

^a^CONNEX-1 (NCT04846868), CONNEX-2 (NCT04846881), and CONNEX-3 (NCT04860830). CONNEX-1, -2, and -3 trials all included a follow‑up (CONNEX-X, NCT05211947) that is not depicted here.

Ext, extension; MATRICS, Measurement and Treatment Research to Improve Cognition in Schizophrenia; MCCB, MATRICS Consensus Cognitive Battery; R, randomization; S, screening.

## Figure S2. Box plot to compare the distribution of scores for VRFCAT overall T-score and CGI-S known groups at baseline

CGI-S, Clinical Global Impression of Severity; VRFCAT, Virtual Reality Functional Capacity Assessment Tool.

## Figure S3. Box plot to compare distribution of scores for VRFCAT T-score and CGI-S known groups at Week 12

CGI-S, Clinical Global Impression of Severity; VRFCAT, Virtual Reality Functional Capacity Assessment Tool.

## Figure S4. Box plot to compare distribution of scores for VRFCAT T-score and CGI-S known groups at Week 26

CGI-S, Clinical Global Impression of Severity; VRFCAT, Virtual Reality Functional Capacity Assessment Tool.

# Supplementary References

Harvey PD, Horan WP, Atkins AS, et al. Factor structure of cognitive performance and functional capacity in schizophrenia: Evidence for differences across functional capacity measures. *Schizophr Res*. 2020;223:297-304. doi: 10.1016/j.schres.2020.08.010.

Horan WP, Depp CA, Hurst S, et al. Qualitative Analysis of the Content Validity of the Virtual Reality Functional Capacity Assessment Tool (VRFCAT) in Schizophrenia: A Multi-Stakeholder Perspective. *Schizophr Bull Open*. 2023;4(1):sgad012. doi: 10.1093/schizbullopen/sgad012.

Keefe RSE, Davis VG, Atkins AS, et al. Validation of a Computerized test of Functional Capacity. *Schizophr Res*. 2016;175(1-3):90-96. doi: 10.1016/j.schres.2016.03.038.

Langevin N, Shukla A, Bogoeva N, et al. The Lived Experience of Cognitive Impairment Associated With Schizophrenia: A Conceptual Model. *Schizophr Bull Open*. 2026;7(1):sgag010. doi: 10.1093/schizbullopen/sgag010.

Nahum M, Lee H, Fisher M, et al. Online Social Cognition Training in Schizophrenia: A Double-Blind, Randomized, Controlled Multi-Site Clinical Trial. *Schizophr Bull*. 2021;47(1):108-117. doi: 10.1093/schbul/sbaa085.

Ventura J, Welikson T, Ered A, et al. Virtual reality assessment of functional capacity in the early course of schizophrenia: Associations with cognitive performance and daily functioning. *Early Interv Psychiatry*. 2020;14(1):106-114. doi: 10.1111/eip.12831.
